# EQUANU: Equality in Societal and Professional Recognition of Nurses—A Cross-Sectional Study on Societal and Professional Recognition of European Nurses

**DOI:** 10.1155/jonm/7466527

**Published:** 2025-02-26

**Authors:** Elyne De Baetselier, Luis Manuel da Cunha Batalha, José Miguel Sousa Pedro Seguro, Nienke E. Dijkstra, Vigdis Abrahamsen Grøndahl, Jana Heczková, Ann Karin Helgesen, Rebeka Lekše, Manuel Lillo-Crespo, Alba Malara, Laura Petraglia, Andrea Pokorná, Mirko Prosen, Styliani Tziaferi, Tinne Dilles

**Affiliations:** ^1^Centre for Research and Innovation in Care, NuPhaC, Faculty of Medicine and Health Sciences, University of Antwerp, Antwerp, Belgium; ^2^Health Sciences Research Unit: Nursing (UICISA: E), Nursing School of Coimbra (ESEnfC), Coimbra, Portugal; ^3^Sanfil Medicina, Coimbra, Portugal; ^4^Research Group Care for the Chronically Ill, University of Applied Sciences Utrecht, Utrecht, The Netherlands; ^5^Faculty of Health, Welfare and Organization, Ostfold University College, Halden, Norway; ^6^Na Homolce Hospital, Prague, Czech Republic; ^7^Faculty of Health Sciences, University of Primorska, Izola, Slovenia; ^8^Department of Nursing, Alicante University, Alicante, Spain; ^9^ANASTE Humanitas Foundation, Rome, Italy; ^10^Department of Health Sciences, Faculty of Medicine, Masaryk University, Brno, Czech Republic; ^11^Department of Nursing, Laboratory of Nursing Research and Care, University of Peloponnese, Tripolis, Greece

**Keywords:** motivation, nurses, professional autonomy, professional practice, respect, social comparison, social identification, social status, work environment

## Abstract

**Background:** Despite trends towards greater professionalisation of the nursing profession and an improved public image in certain countries, studies also show that large proportions of the public still do not fully appreciate nurses' competencies. Mapping differences in the societal and professional recognition of nurses allows for benchmarking among countries.

**Aim:** To investigate the level of societal recognition of the nursing profession in nine European countries, and the level of professional recognition perceived by European nurses themselves; to compare levels of recognition between countries; and to identify influencing factors.

**Methods:** A cross-sectional study was conducted. Through an online survey, the study surveyed both the general public and nurses from various healthcare settings across nine countries between December 2022 and June 2023. The instrument used was a combination of self-developed questions on societal and professional recognition, the Work Motivation Scale and an adapted version of the Multidimensional Work Motivation Scale. Data were analysed using SPSS v.29.0, with socioeconomic prestige scores for the public and work environment/work motivation scores for nurses calculated accordingly.

**Results:** A total of 1618 adult citizens and 2335 nurses participated. The public predominantly characterised nurses with attributes such as friendliness, warmth, empathy and compassion. The mean socioeconomic prestige score assigned to nurses was 7.2/10 (SD 1.9), with Portugal having the highest score (*M* 7.5/10, SD 2.0) and Norway the lowest (*M* 5.8/10, SD 1.4; *p* < 0.001). Professional recognition experienced by nurses was generally low (54% indicated rather low, 17% very low). Slovenia, the Netherlands and Belgium had slightly higher mean scores (all *M* 1.4/3) compared to other countries (*p* < 0.001). High professional recognition could be predicted for 33% by work environment score (OR = 1.21; 95% CI [1.19–1.24]), work motivation score (OR = 1.02; 95%CI[1.01–1.02]), expertise outside the hospital (OR = 1.57; 95% CI [1.25–1.97]) and work experience (OR = 1.01; 95% CI [1.00–1.02]) corrected for country.

**Conclusion:** The study highlights the need for targeted interventions to improve the professional and public image of the nursing profession while addressing disparities in professional recognition between countries. Longitudinal studies are recommended to monitor changes in public perception and professional recognition among nurses.

## 1. Introduction

In 2018, the International Council of Nurses and the World Health Organisation (WHO) launched the 3-year programme *Nursing Now* to enhance global health by elevating the status and profile of nurses worldwide. As nurses comprise half of the global health workforce, their empowerment is pivotal to improving health outcomes on a global scale [1, 2].

Globally, the nursing profession is characterised by the continuous development of knowledge and skills. Despite this trend towards greater professionalisation and an improved public image in certain countries, studies show that a large proportion of the public still does not fully appreciate the competencies of nurses [3, 4]. Indeed, the public may not completely understand the scope of responsibilities and the level of skills required in nursing. The perception that nursing primarily involves basic caregiving tasks can diminish the profession's appeal and fail to highlight the critical role nurses play in patient care [5]. To illustrate, the role of nurses in functions such as adverse drug reaction (ADR) monitoring often goes unnoticed [6]. Managing the therapeutic and adverse effects of medication is a vital responsibility for nurses, requiring them to detect and manage ADRs to ensure patient safety [7]. Among many other tasks, ADR monitoring demands a high degree of expertise and vigilance, demonstrating nurses' advanced clinical skills and their significant impact on patient outcomes [8]. Additionally, nurses often face challenging working conditions, including long hours, high patient-to-nurse ratios, and physically and emotionally demanding workloads, which contribute to a negative perception of the profession. Moreover, how nurses are portrayed in the media can influence public perceptions of their roles, the recruitment of new nurses and the profession's overall identity [3, 9]. Media representations that focus on stereotypes can perpetuate an undervalued or negative image of nursing [4, 10]. Furthermore, nurses' self-image appears to partly shape this public image. Low self-esteem among some nurses, combined with their limited presence in public debates, contributes to insufficient social recognition of their profession [3].

Partly as a result of this negative public image in some countries, healthcare institutions experience difficulties in recruiting and keeping skilled nursing professionals [11, 12]. Therefore, improving the public image of the nursing profession is essential for attracting and retaining qualified individuals who are crucial to delivering high-quality patient care. Mitigating negative influences on the public perception of the profession necessitates coordinated efforts among healthcare institutions, educational organisations, professional associations, governmental bodies and media stakeholders.

Recognition is defined as a multifaceted concept encapsulating the essence of being seen, heard, valued and considered. In essence, it involves acknowledging an individual's presence, appreciating their contributions and expressing approval for the efforts [13, 14]. Two types of recognition can be defined in the context of this study: (1) societal recognition refers to the acknowledgement of nurses and the nursing profession by the general public [15, 16]; (2) professional recognition involves nurses themselves acknowledging the nursing profession and its competencies [15, 17].

While studies have examined public and professional perceptions of nurses in individual countries, few have explored these issues on a broader European scale, comparing multiple nations simultaneously. Additionally, limited research has addressed how societal and professional recognition interact or identified the factors driving these disparities. The lack of comprehensive, cross-national data on nurse recognition creates a significant knowledge gap, limiting our ability to design targeted interventions that address the challenges faced by nurses across Europe.

Therefore, the aims of this study were (a) to investigate the level of societal recognition of the nursing profession in nine different European countries, (b) to investigate the level of professional recognition perceived by European nurses themselves, (c) to compare levels of recognition between countries and (d) to identify factors that influence nurse recognition (from both societal and professional viewpoints).

Consequently, the practical purpose of the study is to inform and guide strategies to enhance the recognition and status of the nursing profession across Europe. Furthermore, mapping differences in nurse recognition will provide a benchmark between countries. This can empower nurses in countries with lower recognition levels to strive for a greater recognition, potentially improving the quality of care. Additionally, increased equality may facilitate labour mobility for European nurses.

## 2. Materials and Methods

### 2.1. Study Design

In a quantitative cross-sectional survey conducted across nine European countries, members of the public and nurses were invited to complete an online structured questionnaire addressing the societal and professional recognition of nurses. The design and method were chosen as the most effective approach to engage a diverse range of participants across multiple countries. This report presents findings from the first year of a 9-year longitudinal study aimed at examining equality in societal and professional recognition of nurses (hereinafter abbreviated as EQUANU).

### 2.2. Participants and Setting

Respondents from nine European countries participated in the study: Belgium, Czech Republic, Greece, Italy, the Netherlands, Norway, Portugal, Slovenia, and Spain. The selection of these countries was primarily based on the availability of researchers willing to contribute, along with considerations of geographical distribution and diversity in healthcare systems.

To assess societal appreciation for the nursing profession, a convenience sample of the general public was recruited, with all adult citizens (aged 18 years or older) eligible to participate. For professional recognition, a convenience sample of nurses was selected. Nurses were approached through professional organisations and healthcare institutions in the participating countries to ensure a diverse and comprehensive representation of the ‘European' nurse, encompassing geographical variation and a range of healthcare settings. Eligibility criteria for nurses included those qualified at Levels 4–8 of the European Qualification Framework (EQF) [18]. It is important to note that not all levels of training are available in every country. Both nurses actively involved in patient care and those in managerial roles were eligible, regardless of their employment rate. However, student nurses, retired nurses and nurses on long-term sick leave (> 6 months) were excluded from participation.

A target sample size of 100–300 nurses and an equal number of adult citizens per country were set to provide a representative overview of societal and professional recognition at the country level. Smaller sample sizes per country were permitted to enhance the overall representation at the European level.

The Work Motivation Scale, adapted from the Multidimensional Work Motivation Scale (MWMS) [19], originally consisted of 20 questions around work efforts, scored on a 7-point scale (‘not at all' to ‘completely'). For this study, six items were removed: five based on content validation (as detailed later) and one for reliability. Cronbach's alpha for the adapted scale was 0.83. The work motivation sum score ranged from 0 to 84, with higher scores indicating greater motivation.

The questionnaires were developed collaboratively by the EQUANU team, a European group of 13 nurse researchers and two physician researchers from nine countries. The coordination and initial survey design were led by the Belgian researchers (EDB and TD). Researchers from the other countries contributed to refining the questionnaires, assessing feasibility and acceptability, and ensuring validity and accurate translation of the items. Following the development of the English versions, the questionnaires underwent validation for face and content validity. This process involved 15 researchers across the participating countries, with one or two researchers representing each country. Each item was rated on a 4-point Likert scale ranging from ‘not relevant' to ‘somewhat irrelevant', ‘somewhat relevant' and very relevant. Items with an Item-Content Validity Index (I-CVI) below 0.8 were excluded from the assessment [20]. As a result, 12 items were removed, leaving 68 remaining items for societal recognition and 56 items for professional recognition. Both survey sections are included in the Supporting Information of this article.

The questionnaire was subsequently translated into the official languages of all participating regions to ensure respondents could complete it in their native language. Local researchers, serving as contact persons in each country, oversaw the translation process (forward–backward translation) to ensure accuracy and maintain high-quality standards. Finally, before starting data collection, five pilot questionnaires were conducted, resulting in no further adjustments.

### 2.3. Survey Development

To investigate the level of societal recognition of the nursing profession and the level of professional recognition perceived by European nurses themselves, we developed two separate surveys. Both survey instruments can be found in the Supporting Information:

a. Societal recognition: Respondents provided general demographic data, assessed their experience with nursing care and rated nurses' characteristics by selecting between opposing traits (5-point scale). Societal recognition was rated on a 4-point scale (from ‘lower than it should be' to ‘higher than it should be'). Respondents also rated various professions, including nursing, on a 10-point socioeconomic prestige scale (1 = lowest). Additionally, factors influencing personal recognition of nurses were evaluated using a 5-point Likert scale (from ‘very negative' to ‘very positive'). Respondents were provided with clear instructions on how to answer questions accurately, with reminders that they should focus on recent experiences with nurses, reducing the risk of recall errors.
b. Professional recognition: The questionnaire assessed perceived professional recognition, work environment and work motivation. Professional recognition was rated using a 4-point Likert scale (very low to very high). Work environment was measured with 15 self-developed statements, rated on a 4-point Likert scale (completely disagree to completely agree), with a sum score range of 15–60 (Cronbach's alpha = 0.85).

### 2.4. Data Collection

Between December 2022 and June 2023, each of the nine EQUANU partners (one research team per country, comprising researchers who joined the project based on their intrinsic motivation) implemented a dissemination strategy tailored to their local context. These strategies were designed around the available resources, such as existing organisations, professional nurse associations, healthcare facilities and private networks within their respective countries. A Qualtrics weblink to the survey section on professional recognition was sent by email as part of this dissemination process, with each partner distributing the link to their respective organisations or individual respondents [21]. For the societal recognition part, a Qualtrics weblink was also distributed to eligible respondents via email. Additionally, people were approached in public spaces like streets, shops, stations and parking areas, as well as waiting rooms or entrance halls of healthcare institutions, allowing the inclusion of patients, visitors, informal carers and all types of care providers. Furthermore, the snowballing technique was employed to increase the sample size in the web survey by asking participants to share the URL link with others. Each country received regular updates on the number of participants.

### 2.5. Data Analysis

Data were analysed using the IBM SPSS Statistics version 29.0. Individuals who did not meet the eligibility criteria but accidently received the survey link were excluded from the final sample. A two-sided level of significance of 0.05 was applied. Discontinuous data were described using *n* values and percentages, while continuous data were summarised with the mean (*M*) and standard deviation (SD). The normality of the distribution was assessed using absolute values of skewness and kurtosis due to the large sample size [22]. All data were found to be normally distributed.

The clustering process of opposite characteristics of nurses (e.g., cold–warm; follower–leader) involved grouping responses from a 5-point scale into three categories: leaning towards one characteristic (scores 1–2), neutral (score 3) and leaning towards the opposite characteristic (scores 4–5). Furthermore, we created sum scores for work environment and work motivation, after recoding all items in the same direction and conducting reliability analyses using Cronbach's alpha. For both societal and professional recognition, we explored relationships among demographics, employment, subgroups with differing opinions or beliefs, work environment score and work satisfaction score. To evaluate statistical significance between two groups, independent *T* tests were used, while one-way ANOVA tests were applied for comparisons involving more than two groups (e.g., countries). Pearson's R tests were used to assess correlations between continuous variables. Finally, we performed a multiple linear regression to predict socioeconomic prestige scores and a multiple logistic regression to predict high professional recognition, both using the enter-method. Socioeconomic prestige scores for Slovenia were excluded from all analyses due to a mistranslation in the question, which resulted in inconclusive results.

### 2.6. Ethical Considerations

The Ethics Committee of the University Hospital Antwerp and the University of Antwerp approved the study design (Registration number: B3002022000104). Depending on local regulations, in two countries, additional approval from local organisations was obtained: Ethics Committee of the Health Sciences Research Unit—Nursing of the Nursing School of Coimbra, Portugal (Registration number: P950_03_2023), and Ethics Committee of the Faculty of Medicine of Masaryk University, Czech Republic (Registration number: MU-IS/88967/2023/2000152/LF). All respondents received information on the purpose and design of the study. Before the questionnaire could be started, all respondents had to indicate they had read the study information and consented to participate. Data were collected anonymously to ensure privacy, and participants were assured that their responses would remain confidential, reducing the pressure to provide socially desirable answers.

## 3. Results

### 3.1. Research Population

A total of 3981 respondents participated: 1618 in the societal recognition part, 2335 in the professional recognition part.

The general public in this study comprised 70% women, with a mean age of 42 years (SD 14). More than 90% were professionally active, and 59% had performed shift work either currently or in the past. Almost one-fifth of the sample (17%) had experience as a nurse, and respondents reported having an average of two friends or relatives (range 0–50) employed as nurses. Additionally, 78% had previously received nursing care.

The 2335 nurses surveyed about professional recognition were 87% female, with a mean age of 42 years (SD 12) and an average of 19 years of work experience (SD 12). The majority of the nurses had an educational level of 6 (68%), most expertise in hospital care (76%), and were currently employed in clinical practice (76%). Collaboration with other professionals was primarily with 1–4 other nurses (50%), 1–4 physicians (70%) and most reported not collaborating with pharmacists (60%) on a daily basis.  Table 1 shows the population characteristics for both parts of the study.

### 3.2. Societal Recognition of European Nurses

**Figure 1:**
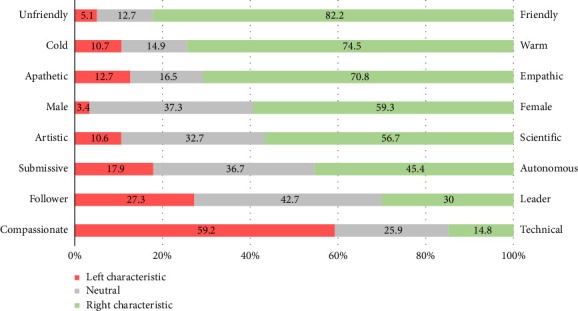
Characteristics attributed to nurses by the general public (*n* = 1603).

**Figure 2:**
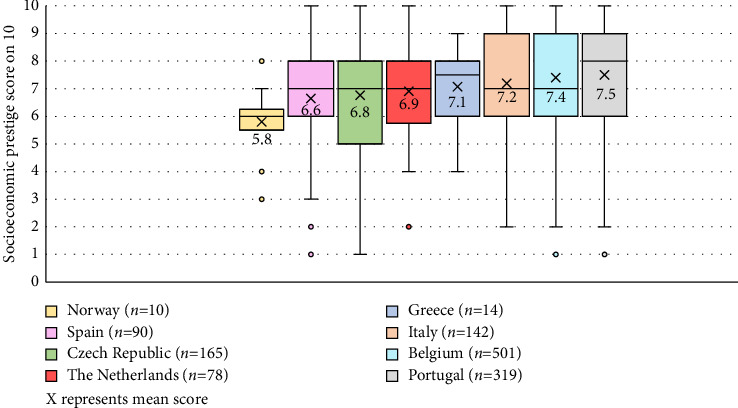
Comparison of the general public's socioeconomic prestige scores for nurses (on 10) between eight European countries (*p* < 0.001).

When showing the general public opposite characteristics about the nursing profession, nurses were predominantly characterised as friendly (82%), warm (75%), empathic (71%), female (59%), scientific (57%) and compassionate (59%). Autonomy (45%), leadership (30%) and technical expertise (15%) were least attributed to nurses (Figure 1). Socioeconomic prestige scores for 12 professions showed that nurses were allocated a mean score of 7.2/10 (SD 1.9), which was lower than physicians (*M* 8.8/10, SD 1.1), air pilots (*M* 8.3/10, SD 2.0), lawyers (*M* 7.9/10, SD 1.9) and engineers (*M* 7.6/10, SD 1.8). In contrast, hairdressers and bus drivers received lowest scores (respectively, *M* 5.4/10, SD 2.1 and *M* 5.3/10, SD 2.3; Table 2). A comparison between countries showed a significant difference, with Portugal having the highest mean score for nurses' socioeconomic prestige (*M* 7.5, SD 2.0) and Norway having the lowest mean score (*M* 5.8, SD 1.4; *p* < 0.001; Figure 2).

The majority of the respondents found the level of societal recognition of nurses in their country lower than it should be (63%). The attractiveness of the nursing profession was considered (very) low by 57% of the public. Questioning the attractiveness before and after the COVID-19 pandemic gave mixed results: 40% of the people thought the profession was more attractive than before, compared to 46% considering it less attractive. Most of the public disagreed that nursing is an independent profession where nurses can make decisions themselves (63%). A considerable proportion (39%) considered the execution of physicians' assignments as the main task of nurses.

The general public reported that their recognition of nurses was negatively influenced by nurses' workload (52%), the number of nurses (48%) and their salary (46%). In contrast, recognition was positively influenced by the societal importance of nursing (77%), interprofessional collaboration (70%), nurses' competencies (67%) and their job security (60%). Finally, most respondents would allow their children to study nursing (96%), although less people would encourage them to do so (62%). We compared the latter percentages with the subsample of nurses (both nurses that participated in the survey about societal recognition (*n* = 220) and those in the professional recognition survey (*n* = 1981)) and found that less nurses would allow (87%) and encourage (54%) their children to study nursing.

Significantly lower mean scores for the socioeconomic prestige of nurses were found when comparing the following groups in the public sample: having experience in healthcare (*M* 6.9, SD 2.2) versus respondents without experience as a healthcare worker (*M* 7.4, SD 2.0; *p* < 0.001), having negative experiences with nurses (*M* 6.0, SD 1.8) versus positive experiences (*M* 7.3, SD 1.9; *p* < 0.001), having low respect for nurses (*M* 6.4, SD 2.1) versus high respect (*M* 7.3, SD 1.9;*p* < 0.001), perceiving the profession as little attractive (*M* 6.9, SD 1.9) versus high attractivity (*M* 7.7, SD 1.8; *p* < 0.001) and disbelief in academic degrees for nurses (*M* 6.8, SD 2.3) versus believing nurses can have academic degrees such as a master or PhD degree (*M* 7.3, SD 1.9; *p* = 0.036). Furthermore, significant, although very small correlations were seen between the public's mean socioeconomic prestige score for nurses and age (*r* = 0.1; *p* < 0.001) and the public's perceived knowledge about nurses' job content (*r* = −0.1; *p* = 0.002). No relation was seen between the socioeconomic prestige score and gender (*p* = 0.498), number of friends/relatives that received nursing care the past year (*p* = 0.727) or that worked as a nurse (*p* = 0.897).

Finally, in a multiple linear regression, societal recognition (by means of socioeconomic prestige scores of the general public) could be predicted for 14% by finding the nursing profession attractive (*B* = 0.82, *p* < 0.001), having positive personal experience with nurses (*B* = 1.07, *p* = 0.002), thinking society appreciates the own job (*B* = 0.65, *p* < 0.001), being a nurse (*B* = −0.80, *p* < 0.001) and having most experience in community care (*B* = 0.55, *p* = 0.025) corrected for country.

### 3.3. Professional Recognition of European Nurses

#### 3.3.1. Nurses' Views on Their Work Environment

**Figure 3:**
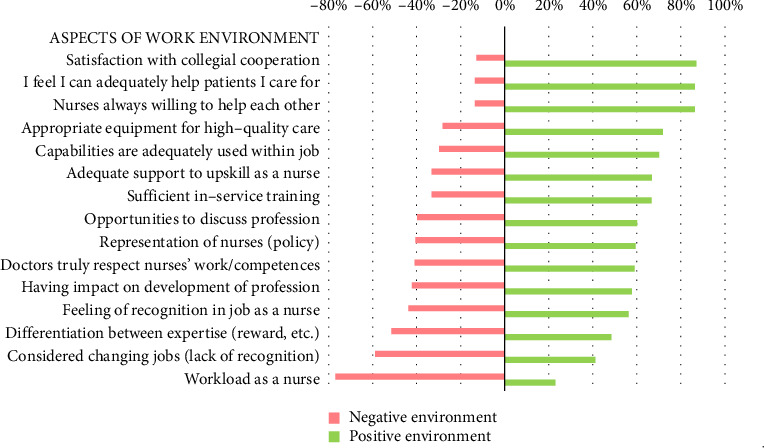
Percentages of nurses' perceiving aspects of their work environment as negative or positive (*n* = 2325).

Most nurses reported experiencing a positive work environment. They were most satisfied with the cooperation with their colleagues (87%), followed by their ability to adequately help the patients they cared for (86%), the willingness of nurses on the ward to assist one another (86%) and the provision of appropriate equipment, supplies and technology to optimise the efficient delivery of high-quality nursing care (72%) (Figure 3). The mean work environment score was 40 out of 60 (SD 6.6), reflecting a generally positive perception of the work environment, though with some room for improvement. The lowest mean scores were observed in Greece, Norway and Portugal (all *M* 36/60), while the highest were recorded in the Netherlands and Belgium (*M* 42.5/60 and *M* 43.5/60, respectively; *p* < 0.001; Table 3).

#### 3.3.2. Nurses' Main Reasons for Investing Efforts in Their Job

**Figure 4:**
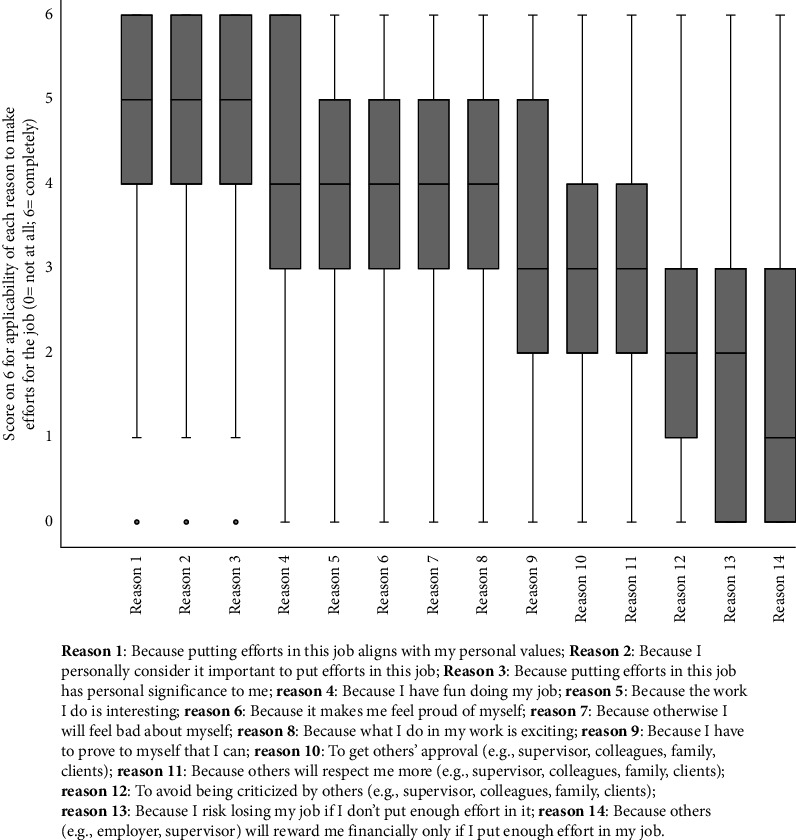
Nurses' main reasons for investing efforts in their job (*n* = 2332).

The nurses' mean work motivational score was 48.7/84 (SD 12.0), with Norway having the lowest motivation (*M* 34.4/84, SD 14.7) and Spain having the highest motivation (*M* 53.6, SD 10.8; *p* < 0.001; Table 3). The three main reasons for nurses to make efforts or get involved in their job were (1) putting efforts in their job aligned with their personal values (*M* 4.7/6; SD 1.3, *Mdn* 5/6); (2) considering it important to put efforts in their job (*M* 4.6/6; SD 1.3, *Mdn* 5/6); and (3) putting efforts in their job had personal significance to them (*M* 4.5/6; SD 1.4, *Mdn* 5/6).  Figure 4 shows all the reasons that were presented to and rated by nurses for investing efforts in their job.

#### 3.3.3. Professional Recognition Perceived by Nurses

**Figure 5:**
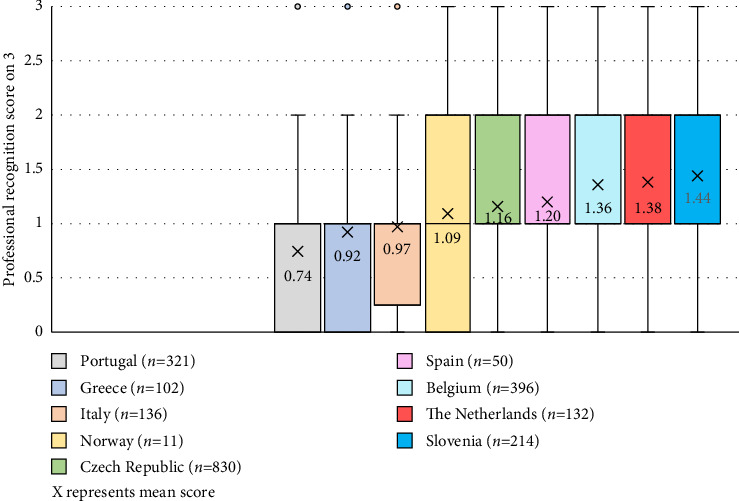
Comparison of professional recognition scores (on 3) between nine European countries (*p* < 0.001).

Most nurses perceived their professional recognition as rather low (54%) or very low (17%). Professional recognition depended on the healthcare settings where nurses reported to have most expertise, with the lowest perceived recognition by hospital care nurses (*M* 1.1/3, SD 0.7) and the highest by mental healthcare nurses (*M* 1.4/3, SD 0.7; *p* = 0.006). Also, nurses with current employment in policy perceived higher recognition (*M* 1.4/3, SD 0.7) compared to other nurses (1.2/3 in education and research; 1.1/3 in clinical practice; *p* < 0.001). Work environment was moderately correlated to professional recognition (*r* = 0.5, *p* < 0.001), while age (*r* = 0.1, *p* < 0.001), years of work experience (*r* = 0.1; *p* < 0.001) and work motivation (*r* = 0.2; *p* < 0.001) had weak, positive correlations. Furthermore, comparisons between countries showed differences in professional recognition (*p* < 0.001), with Slovenia, the Netherlands and Belgium having slightly higher mean scores (respectively, 1.44, 1.40 and 1.36/3) compared to other countries (Figure 5). Professional recognition was not related to gender (*p* = 0.575) or highest educational level of the nurse (*p* = 0.409).

Finally, in a multiple regression model (rather/very), high professional recognition could be predicted for 33% by having most expertise outside hospital care (OR = 1.57; 95%CI[1.25–1.97]), the work environment score (OR = 1.21; 95%CI[1.19–1.24]), the work motivation score (OR = 1.02; 95%CI[1.01–1.02]) and the years of work experience (OR = 1.01; 95%CI[1.00–1.02]) corrected for country.

## 4. Discussion

The ‘EQUANU' findings shed light on disparities in societal and professional recognition, offering valuable insights for policymaking and fostering a more equitable nursing landscape The professional recognition aspect of the study delved into nurses' work environments and motivations. The positive work environment reported by most nurses, particularly the satisfaction with cooperation and the provision of necessary resources, suggests an atmosphere that supports collaboration, productivity and overall job satisfaction. However, the study acknowledged a perception among nurses that their professional recognition is generally low, emphasising a need for interventions to enhance their status. Finally, the association among the work environment, work motivation and professional recognition emphasises the interconnectedness of these factors.

Results for societal recognition revealed nuanced perspectives among the general public. Nurses were predominantly characterised with attributes such as friendliness, warmth, empathy and compassion and not with autonomy, leadership and technical expertise. Also, the mean socioeconomic prestige score assigned to nurses indicated room for improvement compared to other professions like physicians, air pilots, lawyers and engineers. In contrast, the findings from the recent Gallup poll in the United States, which assessed the honesty and ethical standards of professions, placed nurses at the very top for the 23rd consecutive year [23]. With three-quarters of the respondents rating nurses' honesty and ethics as ‘very high' or ‘high', nursing was ranked above all other professions, including physicians, lawyers and engineers. This stark difference highlights the multidimensional nature of recognition, where ethical trustworthiness and socioeconomic prestige do not always align.

The positive characterisation of nurses in our study (friendly, warm, empathetic) aligns with numerous studies, highlighting the caring nature of nursing as a profession [24, 25]. However, it is regrettable that our findings, from a European perspective, indicate that attributes such as technicity, autonomy and leadership are less frequently recognised. While several studies collectively affirm that nurses possess technical expertise, exercise professional autonomy and engage in leadership roles within healthcare settings [26, 27], our study—focusing on the general public's perception—remains rooted in the ‘softer' image of nurses as primarily caring and empathic professionals. Our results showed that most of the public disagreed that nursing is an independent profession where nurses can make decisions themselves and also a large part considered the execution of physicians' assignments as the main task of nurses. Indeed, as other studies demonstrate, society still seems to be unaware of nurses' extensive responsibilities and competencies [5, 28]. The media likely play a substantial role in reinforcing this perception [3, 11, 29]. Despite strong evidence demonstrating that nurses are far more than the assisting hand of physicians, the public image of nursing risks remaining entrenched in historically constructed narratives unless media representations evolve. To enhance societal recognition of nurses, it is essential for media outlets to actively highlight and disseminate findings from studies that portray the multifaceted and highly skilled nature of nursing.

By emphasising the complexity and importance of nurses' responsibilities, public awareness of their advanced nursing skills can be improved, thereby enhancing the professional recognition of nursing. The study's exploration of factors influencing recognition, including societal importance, interprofessional collaboration, nurses' competencies and job security, provides a comprehensive understanding of the dynamics shaping public opinion [2, 5, 30–32].

Comparing recognition across European countries revealed significant variations in the perceived socioeconomic prestige of nurses, with Norway ranking the lowest and Portugal the highest. However, limited sample sizes in some countries make it challenging to draw definitive conclusions at the country level. We therefore recommend increasing the sample size before conducting further analyses to explore statistical differences between countries.

### 4.1. Strengths and Limitations

The EQUANU study makes a valuable contribution to the ongoing dialogue about the recognition of nurses in European countries [3, 4, 33, 34]. By addressing both societal and professional dimensions, the study provides a comprehensive view on the challenges and opportunities faced by the nursing profession. These insights can guide policymakers, healthcare organisations and educators in developing strategies that enhance the status and recognition of nurses, fostering a more equitable and supportive environment for this crucial healthcare workforce. Despite the limited number of participants at the national level in some countries, the overall sample size was satisfactory and provided interesting insights into current societal and professional recognition of European nurses.

This Internet survey had limitations. The inclusion or exclusion of countries and respondents was determined by whether they agreed or declined to participate in the study. This self-selected sample with an unknown response rate might have led to a distortion of the results due to only the most motivated people participating. Moreover, the impact of having nearly half of participants with healthcare experience in our sample for societal recognition should not be overlooked. We previously highlighted the significant difference between participants with and without healthcare backgrounds in terms of socioeconomic prestige scores. However, it is plausible that other findings may have been affected as well. In our longitudinal EQUANU study, we are committed to addressing and rectifying this imbalance. Finally, we acknowledge that our findings represent perceptions in each individual cultural context and are not validated against direct observations or correlated with any outcomes.

### 4.2. Implications and Recommendations

The results of the EQUANU study have several implications for nursing practice, education and policy. Firstly, the identified disparities in societal and professional recognition call for targeted interventions to improve the public image of nursing. Initiatives should focus on educating the public about the evolving roles and responsibilities of nurses and highlighting their contributions to healthcare [4, 32].

Additionally, efforts to address the perceived low professional recognition among nurses should consider enhancing work environments [35], recognising the diversity of roles within the profession and hierarchy in nursing teams, and promoting interprofessional collaboration [31]. Tailored strategies for different healthcare settings and roles within nursing may be necessary to ensure equitable recognition.

The study's cross-sectional nature provides a snapshot of the current situation, but longitudinal studies could offer insights into the evolution of societal and professional perceptions over time. According to Blau, Sela and Grinberg, the public's opinion and perception of the nursing profession have become more positive since the COVID-19 pandemic, compared to other professions [36]. It is, therefore, important to continue investigating which key factors contributed to this change and perception and to develop strategies aimed at maintaining and improving the positive image of nursing among the public on the long term. The EQUANU study is therefore a first step in a 9-year longitudinal research plan to explore the impact of cultural, contextual and time factors on the recognition of nurses.

## 5. Conclusion

This study investigated the level of societal recognition of the nursing profession in nine European countries, as well as the level of professional recognition perceived by European nurses themselves. On the one hand, the public viewed nurses as friendly, warm and empathic individuals but attributed limited traits of autonomy, leadership or technical expertise to the profession. This perception was further reflected in the relatively low socioeconomic prestige of nurses compared to that of other professions. On the other hand, while nurses generally held a positive view of their work environment, a significant proportion still reported feeling a lack of professional recognition, particularly those working in hospital settings. Although this study offers valuable insights into the current situation, future longitudinal research is essential to explore the evolution of these perceptions over time, particularly considering the increased public appreciation for nursing since the COVID-19 pandemic. The identified disparities in societal and professional recognition highlight the need for targeted interventions to improve the public image of nursing. Efforts by policymakers, healthcare leaders, educators and professional nursing organisations should focus on public education, improving work environments and fostering interprofessional collaboration.

## Figures and Tables

**Table 1:** Population characteristics.

**Societal recognition (*n* = 1618)**	

*Country*, *n* (%)	
Belgium	515 (31.8)
Portugal	319 (19.7)
Slovenia	284 (17.6)
Czech Republic	165 (10.2)
Italy	142 (8.8)
Spain	90 (5.6)
The Netherlands	79 (4.9)
Greece	14 (0.9)
Norway	10 (0.6)
*Gender*, *n* (%)	
Female	1132 (70.0)
Male	480 (29.7)
Other/prefer not to say	3 (0.3)
*Age* (years), *M* (SD)	42.3 (14.1)
*Current professional status*, *n* (%)	
Self-employed	621 (46.7)
Employee or civil servant	411 (31.0)
Student worker	78 (5.9)
Unemployed	69 (5.2)
Family worker	55 (4.1)
Combination of jobs	46 (3.5)
Retired	43 (3.2)
*Shift work* (present or past), *n* (%)	912 (58.9)
*Appreciation for own job*, *n* (%)	
Appreciation in society for own job	1070 (68.9)
Appreciation from employer for own job	1148 (75.5)
Monthly wage represents own efforts	658 (43.0)
*Experience as healthcare worker*, *n*(%)	
No	908 (57.5)
Nurse	269 (17.0)
Physician	98 (6.2)
Physiotherapist	157 (9.9)
Nurse assistant	28 (1.8)
Psychologist	15 (0.9)
Pharmacist	9 (0.6)
Speech therapist	5 (0.3)
Ambulance	5 (0.3)
Other	85 (5.4)
If experience in healthcare (*n* = 671^a^), *setting with most experience*, *n* (%)	
Hospital care	255 (53.1)
Residential care	97 (20.2)
Community care	74 (15.4)
Mental healthcare	31 (6.5)
No clinical practice experience	23 (4.8)
If experience in healthcare (*n* = 671^b^), *current employment*, *n* (%)	
Clinical practice	435 (68.7)
Education	108 (17.1)
Policymaking	53 (8.4)
Research	37 (5.8)
*Received nursing care*, *n* (%)	
Never received	342 (21.2)
During one or more hospitalisations	1021 (63.4)
While resided in a healthcare facility	170 (10.6)
At home for a short period	122 (7.6)
At home for more than 3 months	33 (2.0)
Other	67 (4.2)
*People one cares about that received nursing care the last year*	
I know such persons, *n* (%)	1311 (82.9)
Number of persons, median (min–max)	2 (0–50)
*Friends/relatives employed as a nurse*	
I know such persons, *n* (%)	1208 (75.8)
Number of friends, median (min–max)	2 (0–50)

**Professional recognition (*n* = 2335)**	

*Country* (working), *n* (%)	
Belgium	416 (17.8)
Portugal	335 (14.3)
Slovenia	227 (9.7)
Czech Republic	912 (39.0)
Italy	140 (6.0)
Spain	56 (2.4)
The Netherlands	132 (5.7)
Greece	106 (4.5)
Norway	12 (0.5)
*Country* (educated), *n*(%)	
Belgium	405 (17.4)
Portugal	333 (14.3)
Slovenia	231 (9.9)
Czech Republic	896 (38.4)
Italy	126 (5.4)
Spain	58 (2.5)
The Netherlands	131 (5.6)
Greece	104 (4.5)
Norway	11 (0.5)
Other	35 (1.5)
*Gender*, *n* (%)	
Female	2036 (87.2)
Male	294 (12.6)
Other/prefer not to say	6 (0.2)
*Age* (years), *M* (SD)	41.5 (11.8)
*Work experience* (years), *M* (SD)	19.1 (12.4)
*Highest level of nursing education* (EQF^c^), *n* (%)	
Level 4	40 (7.4)
Level 5	87 (16.1)
Level 6	369 (68.2)
Level 7	44 (8.1)
Level 8	1 (0.2)
*Nonmandatory extra education*, *n* (%)	
None	119 (5.3)
< 1 day/year	146 (6.5)
1–2 days/year	430 (19.2)
> 2 d/year	1520 (68.9)
*Healthcare setting with most expertise*, *n* (%)	
Hospital care	1598 (76.2)
Community care	193 (9.2)
Residential care	186 (8.9)
Mental healthcare	86 (4.1)
No clinical practice experience	33 (1.6)
*Current employment*, *n* (%)	
Clinical practice	1984 (85.6)
Education	152 (6.6)
Policymaking	155 (6.7)
Research	27 (1.2)
*Daily collaboration with*, *n* (%)	
*Nurses*: none	136 (5.8)
< 5	1162 (50.0)
5–10	632 (27.2)
> 10	395 (17.0)
*Physicians*: none	100 (4.3)
< 5	1613 (69.8)
5–10	411 (17.8)
> 10	186 (8.1)
*Pharmacists*: none	1391 (60.4)
< 5	869 (37.7)
5–10	29 (1.3)
> 10	13 (0.6)

^a^28.5% of 671 did not answer this question.

^b^5.7% of 671 did not answer this question.

^c^EQF = European Qualification Framework [18].

**Table 2:** Mean socioeconomic prestige scores of the general public from 0 to 10 of 12 different professions in eight countries (*n* = 1317).

Profession	Mean score (SD)
Physician	8.8 (1.5)
Air pilot	8.3 (2.0)
Lawyer	7.9 (1.9)
Engineer	7.6 (1.8)
Nurse	7.2 (1.9)
Teacher	7.0 (1.9)
Police officer	6.9 (2.0)
Electrician	6.2 (2.0)
Musician	5.8 (2.2)
Bookkeeper	5.7 (2.0)
Hairdresser	5.4 (2.1)
Bus driver	5.3 (2.3)

**Table 3:** Perceived work environment and work motivation compared between European nurses.

Work environment scores M (SD); range 15–60		Work motivation scores M (SD); range 0–84	
Belgium (*n* = 339)	43.5 (5.5)	Spain (*n* = 56)	53.6 (10.8)
The Netherlands (*n* = 132)	42.5 (4.4)	Belgium (*n* = 339)	52.1 (10.1)
Spain (*n* = 56)	41.1 (7.5)	Italy (*n* = 140)	50.9 (10.9)
Czech Republic (*n* = 912)	40.8 (6.4)	Slovenia (*n* = 227)	50.0 (11.6)
Slovenia (*n* = 227)	39.9 (6.2)	Czech Republic (*n* = 912)	48.0 (10.7)
Italy (*n* = 140)	39.3 (7.1)	Greece (*n* = 106)	46.9 (10.8)
Portugal (*n* = 335)	36.8 (6.0)	Portugal (*n* = 335)	46.1 (12.4)
Greece (*n* = 106)	36.5 (7.5)	The Netherlands (*n* = 132)	46.0 (10.7)
Norway (*n* = 12)	36.5 (8.3)	Norway (*n* = 12)	34.4 (14.7)

## Data Availability

The data that support the findings of this study are available from the corresponding author upon reasonable request.

## Supporting Information

Supporting Information 1. Survey: The survey consisted of two survey parts: (1) societal recognition and (2) professional recognition. Supporting Information 2. STROBE: Reporting guideline used to prepare and submit this observational study.
