# Supplementary material for: EQUANU: Equality in Societal and Professional Recognition of Nurses—A Cross-Sectional Study on Societal and Professional Recognition of European Nurses
Source: J Nurs Manag. 2025 Feb 26;2025:7466527. doi: 10.1155/jonm/7466527 (PMC11985239; doi:10.1155/jonm/7466527)
Supplement: Supporting Information — Additional supporting information can be found online in the Supporting Information. [file 7466527.f1.docx]

**“EQUANU” PART 1 – for the broad public (18+)**

**Equality in societal and professional recognition of nurses**

Dear participant,

thank you for taking the time to complete this survey, which is conducted in adults of 10 European countries. Its aim is to investigate the public image of nurses. Both healthcare workers and the public are invited to participate. The questionnaire will take approximately 10 minutes to complete.

**Demographical and work-related questions**

1. In what country are you currently living? …………………………………………

2. What is your gender?

0_1_ Male

0_2_ Female

0_3_ Other

0_4_ Prefer not to say

3. What is your age? ………….

4. What is your current professional status?

0_0_ retired

0_1_ unemployed

0_2_ family worker

0_3_ employee

0_4_ self-employed with(out) employees

0_5_ Other: …………………………………..

5. ***(if Question 4 ≠ unemployed):*** are/were you performing shift work? 0_0_ no 0_1_ yes

6. To what extent do you agree with the following statements about the appreciation of your job?

| Statement | Strongly disagree | Disagree | Agree | Strongly agree |
| --- | --- | --- | --- | --- |
| 6a. To my opinion, there is appreciation in society for my job | _1_ | _2_ | _3_ | _4_ |
| 6b. To my opinion, there is appreciation from my employer for my profession | _1_ | _2_ | _3_ | _4_ |
| 6c. To my opinion, my monthly wage accurately represents my efforts | _1_ | _2_ | _3_ | _4_ |

7. Do you have experience as a healthcare worker?

0_0_ No

0_1_ Yes, I am/was a nurse

0_2_ Yes, I am/was a physician

0_3_ Yes, I am/was a pharmacist

0_4_ Yes, I am/was a physiotherapist

0_5_ Yes, I am/was another type of healthcare worker: ……………………

8. ***(if Question 7 ≠ no):*** In which healthcare setting do you have most expertise?

0_1_ community care

0_2_ residential care

0_3_ hospital care

0_4_ mental healthcare

0_5_ no experience in clinical practice

9. ***(if Question 7 ≠ no):*** Which of these best describe your employment at the moment?

0_1_ clinical practice

0_2_ research

0_3_ education

0_4_ policy making

0_5_ no experience in clinical practice

10. From the persons you care about, how many did receive nursing care in the last year? …………

11. How many of your friends or relatives are / were employed as a nurse? ……………

12. What experience do you have with receiving nursing care? (multiple answers possible)? …

0_a_ I have never received nursing care.

0_b_ I have received nursing care during one or more hospitalizations

0_c_ I have received nursing care at home for a short period.

0_d_ I have received nursing care at home for more than three months.

0_e_ I have received nursing care while I resided in a health care facility.

0_f_ Other................................

**Social recognition of European nurses**

13. What are the first three words that come to mind when you think of a nurse.

…………………….….. …………………….…… …………………….…..

14. Five opposite characteristics are shown below. Select for each pair the circle that best fits your opinion about nurses.

14a. Warm 0_1_ 0_2_ 0_3_ 0_4_ 0_5_ Cold

14b. Unfriendly 0_1_ 0_2_ 0_3_ 0_4_ 0_5_ Friendly

14c. Submissive 0_1_ 0_2_ 0_3_ 0_4_ 0_5_ Autonomous

14d. Empathic 0_1_ 0_2_ 0_3_ 0_4_ 0_5_ Apathetic

14e. Scientific 0_1_ 0_2_ 0_3_ 0_4_ 0_5_ Artistic

14f. Follower 0_1_ 0_2_ 0_3_ 0_4_ 0_5_ Leader

14g. Compassionate professional 0_1_ 0_2_ 0_3_ 0_4_ 0_5_ Technical professional

14h. Male 0_1_ 0_2_ 0_3_ 0_4_ 0_5_ Female

Gender

neutral

15.How would you rate the following professions on a 10-point scale of socio-economic prestige? (1/10 is the lowest socio-economic prestige) It is possible to give several professions the same score.

Air pilot …..

Bus driver ..…

Book keeper ..…

Engineer …..

Electrician …..

Hair dresser …..

Lawyer …..

Musician …..

Nurse …..

Physician ..…

Police officer ..…

Teacher …..

16. When you think about the way in which the nurse profession is valued in your country, what is your opinion about this level of societal recognition?

0_0_ Lower than it should be 0_1_ Acceptable 0_2_ Good 0_3_ Higher than it should be

17. Please, indicate for the following statements the answering category that best fits your opinion.

| 17a. My personal experience with nurses is: | Very negative | Negative | Positive | Very positive |
| --- | --- | --- | --- | --- |
| 17b. My satisfaction about nurses’ functioning is: | Very low | Low | High | Very high |
| 17c. My respect for nurses is: | Very limited | Limited | High | Very high |
| 17d. In my opinion, the attractiveness of the nursing profession is: | Very limited | Limited | High | Very high |
| 17e. To my opinion, the attractiveness of the  nursing profession is better than before the COVID pandemic | Not agree at all | Not agree | Agree | Completely agree |
| 17f. To my opinion, the attractiveness of the  nursing profession is worse than before the COVID pandemic | Not agree at all | Not agree | Agree | Completely agree |
| 17g. To my opinion, nursing is an independent profession where nurses can make decisions by themselves | Not agree at all | Not agree | Agree | Completely agree |
| 17h. To my opinion, the main task of nurses is the execution of physicians’ assignments | Not agree at all | Not agree | Agree | Completely agree |
| 17i. In my opinion, the professionalisation of the nursing profession is increasing due to the ever more extensive development of knowledge and skills | Not agree at all | Not agree | Agree | Completely agree |
| 17j. To my opinion, nurses can have academic degrees (such as master level, PhD, …) | Not agree at all | Not agree | Agree | Completely agree |

**Social recognition of European nurses – influencing factors**

18. Indicate for the factors below the level of influence they have on your personal recognition of the nursing profession:

|  | **Level of influence on your personal recognition of nurses** | | | | |
| --- | --- | --- | --- | --- | --- |
|  | **Very negative** influence on your recognition of nurses | **Negative** influence on your recognition of nurses | **No influence** on your recognition of nurses | **Positive** influence on your recognition of nurses | **Very positive** influence on your recognition of nurses |
| 18a. Shift work | _1_ | _2_ | _3_ | _4_ | _5_ |
| 18b. Salary | _1_ | _2_ | _3_ | _4_ | _5_ |
| 18c. Workload | _1_ | _2_ | _3_ | _4_ | _5_ |
| 18d. Career development opportunities | _1_ | _2_ | _3_ | _4_ | _5_ |
| 18e. Job security | _1_ | _2_ | _3_ | _4_ | _5_ |
| 18f. Number of nurses (workforce) | _1_ | _2_ | _3_ | _4_ | _5_ |
| 18g. Nurses’ competences | _1_ | _2_ | _3_ | _4_ | _5_ |
| 18h. Cooperation between nurses and other health professionals | _1_ | _2_ | _3_ | _4_ | _5_ |
| 18i. Societal importance of nursing | _1_ | _2_ | _3_ | _4_ | _5_ |
| 18j. COVID pandemic | _1_ | _2_ | _3_ | _4_ | _5_ |

19. How would you rank the job importance of the following nurses on a 10-point scale (from 0 = very low importance to 10 = highly important)? It is possible to give professions the same score.

| Types of nurses | Importance on 10 |
| --- | --- |
| 19a. Cardiac nurse (nurse who cares for patients with heart diseases) | ……. |
| 19b. Emergency room nurse | ……. |
| 19c. Geriatric nurse (nurse who cares for older adults in nursing homes) | ……. |
| 19d. Intensive care unit nurse | ……. |
| 19e. Mental health nurse (nurse who cares for psychiatric patients) | ……. |
| 19f. Neonatal (newborn) /gynaecological nurse | ……. |
| 19g. Oncology nurse (nurse who cares for cancer patients) | ……. |
| 19h. Paediatric care nurse (nurse who cares for children) | ……. |
| 19i. Community care nurse / family nurse | ……. |

| Statement  In case I would have children, … | Agree | Don’t agree |
| --- | --- | --- |
| 20a. … I would **not allow** them to study nursing | _1_ | _0_ |
| 20b. …I would **discourage** them to study nursing | _1_ | _0_ |
| 20c. … I would **allow them** to study nursing | _1_ | _0_ |
| 20d. … I would **encourage** them to study nursing | _1_ | _0_ |

Q20. Indicate whether you agree or don’t agree with these statements:

**Views on nurses’ job content**

21. To what extent do you consider your knowledge of the nursing job content to be complete?

0_1_ Full knowledge

0_2_ Broad knowledge

0_3_ Limited knowledge

0_4_ Very limited / no knowledge

22. Give your opinion on the extent to which you think the following activities are part of nurses' job content:

|  | **No part** of nurses’ job | **Limited part** of nurses’ job | **Moderate part** of nurses’ job | **Great part** of nurses’ job |
| --- | --- | --- | --- | --- |
| 22a. Care for personal hygiene of patients | _0_ | _1_ | _2_ | _3_ |
| 22b. Placement of infusions | _0_ | _1_ | _2_ | _3_ |
| 22c. Placement of bladder catheters or stomach probes | _0_ | _1_ | _2_ | _3_ |
| 22d. Preventive healthcare (e.g. follow-up children’s growth) | _0_ | _1_ | _2_ | _3_ |
| 22e. Research | _0_ | _1_ | _2_ | _3_ |
| 22f. Evaluation of medication schedule | _0_ | _1_ | _2_ | _3_ |
| 22gi. Prescribing medicines | _0_ | _1_ | _2_ | _3_ |
| 22h. Decision on continuation/discontinuation of medicines | _0_ | _1_ | _2_ | _3_ |
| 22i. Recognition of complications of treatment | _0_ | _1_ | _2_ | _3_ |
| 22j. Clinical reasoning (e.g. making nursing diagnosis, nursing treatment plans, …) | _0_ | _1_ | _2_ | _3_ |

**Follow-up of nurses’ image over a 9-year period**

We are conducting this study from 2022 to 2031 to investigate the evolution of the public image of nurses in Europe. If you would like to be contacted by our research team during the next years of this study (maximum once a year), please provide your contact details below. Your contacts will not be used for other purposes than for inviting you for these follow-up surveys. You can unsubscribe from these invitation emails at any moment.

23. Email address: …………………………..………………………………………………………………………………………………………………….……

**Thank you for your participation in this study!**

**EQUANU PART 2 – only for nurses:**

**Equality in social and professional recognition of nurses**

Dear participant,

Thank you for taking the time to complete this survey, which is conducted in nurses of 10 European countries. Its aim is to gather your views on nurses’ professional recognition. The questionnaire will take approximately 10 minutes to complete.

**Demographical and work-related questions**

1. Are you a nurse? 0_1_ Yes *-> you can continue the survey* 0_0_ No ***-> end the survey here***

2. In what country are you currently working as a nurse? ………………………………………………

3. In what country were you trained as a nurse? ………………………………………………

4. What is your gender?

0_1_ Male

0_2_ Female

0_3_ Other

0_4_ Prefer not to say

5. What is your age? ………………………………………………

6. How many years of work experience do you have in healthcare? ………………………………………………

7. What is your highest level of nursing education? *(translations per country were adapted to the correct wording used in each country, not as “level x”)*

0_3_ level 3 (in some countries)

0_4_ level 4 (in some countries)

0_5_ level 5 (in some countries)

0_6_ level 6

0_7_ level 7

0_8_ level 8

8. On average, how much time a year do you spend non-mandatory extra education?

0_0_ None

0_1_ On average <1 day / year

0_2_ On average 1-2 days / year

0_3_ On average >2 days / year

9. In which healthcare setting do you have most expertise?

0_1_ Community care

0_2_ Residential care

0_3_ Hospital care

0_4_ Mental healthcare

0_5_ No experience in clinical practice

10. Which of these best describe your employment at the moment?

0_1_ Clinical practice

0_2_ Research

0_3_ Education

0_4_ Policy making

11. Which patient population do you care for in the main?

0_1_ Children (0 - 17 years)

0_2_ Adults (18 – 64 years)

0_3_ Older persons (≥ 65 years)

0_4_ More than one age group

0_0_ None

12. On average, how many patients per day are you responsible for as a nurse in your daily practice?

0_1_ 1 - 4

0_2_ 5 - 7

0_3_ ≥ 8

0_0_ Not applicable

13. How many of the following professionals do you work with in your daily clinical practice?

- **Nurses**

0_0_ None

0_1_ <5

0_2_ 5-10

0_3_ >10

- **Doctors**

0_0_ None

0_1_ <5

0_2_ 5-10

0_3_ >10

- **Pharmacists**

0_0_ None

0_1_ <5

0_2_ 5-10

0_3_ >10

14. Read the following statements about your work environment and indicate whether you completely disagree, disagree, agree or completely agree.

| **Statement** | Completely disagree | Disagree | Agree | Completely agree |
| --- | --- | --- | --- | --- |
| 14a. My organisation has a compensation and reward system that differentiates between nurses with different expertise (e.g. clinical expertise, education, qualifications, …) | _1_ | _2_ | _3_ | _4_ |
| 14b. In my organisation, appropriate equipment, supplies and technology are provided to optimize the efficient delivery of high-quality nursing care | _1_ | _2_ | _3_ | _4_ |
| 14c. Nurses are represented on the consultative bodies that determine the policy and functioning of my organisation | _1_ | _2_ | _3_ | _4_ |
| 14d. On my ward, nurses are always willing to help each other with their work | _1_ | _2_ | _3_ | _4_ |
| 14e. I am satisfied with the cooperation between me and my colleagues | _1_ | _2_ | _3_ | _4_ |
| 14f. In my organisation, I have the opportunity to discuss the nursing profession with other healthcare disciplines | _1_ | _2_ | _3_ | _4_ |
| 14g. In my organisation, my capabilities are adequately used within my job as a nurse | _1_ | _2_ | _3_ | _4_ |
| 14h. In my organisation, I am adequately supported to upskill as a nurse | _1_ | _2_ | _3_ | _4_ |
| 14i. In my organisation, sufficient in-service training is provided | _1_ | _2_ | _3_ | _4_ |
| 14j. I feel I can adequately help patients I care for | _1_ | _2_ | _3_ | _4_ |
| 14k. I feel recognised in my job as a nurse | _1_ | _2_ | _3_ | _4_ |
| 14l. I feel I have an impact on the development of the nursing profession for the future | _1_ | _2_ | _3_ | _4_ |
| 14m. In the past 3 months, I have considered changing jobs because of insufficient recognition in my job as a nurse | _1_ | _2_ | _3_ | _4_ |
| 14n. I perceive my workload as a nurse is too high | _1_ | _2_ | _3_ | _4_ |
| 14o. I feel medical doctors truly respect my work and my competences as a nurse | _1_ | _2_ | _3_ | _4_ |

15. To what extent are the following items reasons for you to make efforts/to get involved in your job? For each item, please describe how applicable it is.

| **Reasons to make efforts/to get involved in your job** | Not at all | Not really | A little | Moderate | Strong | Very strong | Completely |
| --- | --- | --- | --- | --- | --- | --- | --- |
| 15a. Because I will get others’ approval (e.g., supervisor, colleagues, family, clients...) | _0_ | _1_ | _2_ | _3_ | _4_ | _5_ | _6_ |
| 15b. Because others will respect me more (e.g., supervisor, colleagues, family, clients...) | _0_ | _1_ | _2_ | _3_ | _4_ | _5_ | _6_ |
| 15c. To avoid being criticized by others (e.g., supervisor, colleagues, family, clients...) | _0_ | _1_ | _2_ | _3_ | _4_ | _5_ | _6_ |
| 15d. Because others (e.g., employer, supervisor...) will reward me financially only if I put enough effort in my job. | _0_ | _1_ | _2_ | _3_ | _4_ | _5_ | _6_ |
| 15e. Because I risk losing my job if I don’t put enough effort in it | _0_ | _1_ | _2_ | _3_ | _4_ | _5_ | _6_ |
| 15f. Because I have to prove to myself that I can | _0_ | _1_ | _2_ | _3_ | _4_ | _5_ | _6_ |
| 15g. Because it makes me feel proud of myself | _0_ | _1_ | _2_ | _3_ | _4_ | _5_ | _6_ |
| 15h. Because otherwise I will feel bad about myself | _0_ | _1_ | _2_ | _3_ | _4_ | _5_ | _6_ |
| 15i. Because I personally consider it important to put efforts in this job | _0_ | _1_ | _2_ | _3_ | _4_ | _5_ | _6_ |
| 15j. Because putting efforts in this job aligns with my personal values | _0_ | _1_ | _2_ | _3_ | _4_ | _5_ | _6_ |
| 15k. Because putting efforts in this job has personal significance to me | _0_ | _1_ | _2_ | _3_ | _4_ | _5_ | _6_ |
| 15l. Because I have fun doing my job | _0_ | _1_ | _2_ | _3_ | _4_ | _5_ | _6_ |
| 15m. Because what I do in my work is exciting | _0_ | _1_ | _2_ | _3_ | _4_ | _5_ | _6_ |
| 15n. Because the work I do is interesting | _0_ | _1_ | _2_ | _3_ | _4_ | _5_ | _6_ |
| 15o. Because I don’t think this work is worth putting efforts into | _0_ | _1_ | _2_ | _3_ | _4_ | _5_ | _6_ |

**Questions about ‘professional recognition’ of European nurses**

Q16. How do you perceive the professional recognition your receive as a nurse?

0_0_ Very low 0_1_ Rather low 0_2_ Rather high 0_3_ Very high

| Statement  In case I would have children, … | Agree | Don’t agree |
| --- | --- | --- |
| 18a. … I would **not allow** them to study nursing | _1_ | _0_ |
| 18b. …I would **discourag**e them to study nursing | _1_ | _0_ |
| 18c. … I would **allow them** to study nursing | _1_ | _0_ |
| 18d. … I would **encourage** them to study nursing | _1_ | _0_ |

Q17. Indicate whether you agree or don’t agree with these statements:

**Follow-up of nurses’ professional recognition over a 9-year period**

We are conducting this study from 2022 to 2031 to investigate the evolution of the professional recognition of advanced nursing roles. If you would like to be contacted by our research team during the next years of this study (maximum once a year), please provide your contact details below. Your contacts will not be used for other purposes than for inviting you for these follow-up surveys. You can unsubscribe from these invitation emails at any moment.

18. Email address: ………………………………………………………………………………………………………………………………………………

**Thank you for your participation in this study!**

# STROBE Statement—checklist of items that should be included in reports of observational studies

**EQUANU: Equality in societal and professional recognition of nurses**

**A cross-sectional study on societal and professional recognition of European nurses.**

|  | | | | Item No. | | Recommendation | | | | Relevant text from manuscript |
| --- | --- | --- | --- | --- | --- | --- | --- | --- | --- | --- |
| **Title and abstract** | | | | 1 | | (*a*) Indicate the study’s design with a commonly used term in the title or the abstract | | | | Cross-sectional study |
|  |  |  |  |  |  | (*b*) Provide in the abstract an informative and balanced summary of what was done and what was found | | | | See methods and results in abstract |
|  | | | | | | |  |  |  |  |
| Background/rationale | | | | 2 | | Explain the scientific background and rationale for the investigation being reported | | | | See introduction |
| Objectives | | | | 3 | | State specific objectives, including any prespecified hypotheses | | | | 1. to investigate the level of societal recognition of the nursing profession in nine different European countries, b) to investigate the level of professional recognition perceived by European nurses themselves, c) to compare levels of recognition between countries, and d) to identify factors that influence nurse recognition (both from societal and professional viewpoint)   No hypotheses. |
|  | | | | | | |  |  |  |  |
| Study design | | | | 4 | | Present key elements of study design early in the paper | | | | First paragraph of methods: quantitative, cross-sectional survey |
| Setting | | | | 5 | | Describe the setting, locations, and relevant dates, including periods of recruitment, exposure, follow-up, and data collection | | | | Setting: 9 European countries, general public & nurses from all HC settings. Dates: between 12/2022 and 6/2023. |
| Participants | | | | 6 | | (*a*) *~~Cohort study~~*~~—Give the eligibility criteria, and the sources and methods of selection of participants. Describe methods of follow-up~~  *~~Case-control study~~*~~—Give the eligibility criteria, and the sources and methods of case ascertainment and control selection. Give the rationale for the choice of cases and controls~~  *Cross-sectional study*—Give the eligibility criteria, and the sources and methods of selection of participants | | | | Part societal recognition: general public, with all adult citizens (aged 18 years or older). Part professional recognition: Level 4-8 nurses were contacted through professional organisations or healthcare organisations. Convenience sample with online survey + people in streets, shops, stations, parking places, waiting rooms or entrance halls of healthcare institutions. |
|  |  |  |  |  |  | ~~(~~*~~b~~*~~)~~ *~~Cohort study~~*~~—For matched studies, give matching criteria and number of exposed and unexposed~~  *~~Case-control study~~*~~—For matched studies, give matching criteria and the number of controls per case~~ | | | | Not applicable in cross-sectional study design. |
| Variables | | | | 7 | | Clearly define all outcomes, exposures, predictors, potential confounders, and effect modifiers. Give diagnostic criteria, if applicable | | | | In section data analysis. Main outcomes: socio-economic prestige score + (high) professional recognition. Main predictors/confounders: demographics, employment, work environment score, work satisfaction score, country. |
| Data sources/ measurement | | | | 8* | | For each variable of interest, give sources of data and details of methods of assessment (measurement). Describe comparability of assessment methods if there is more than one group | | | | Described in section ‘survey development’.  Socio-economic prestige: questioned on 10-point scale. Professional recognition: 1 question, 4-point Likert scale. Work environment: 15 self-developed questions, 4-point Likert scale. Work motivation: based on adapted version of Multidimensional Work Scale. |
| Bias | | | | 9 | | Describe any efforts to address potential sources of bias | | | | Discussion – strengths and limitations |
| Study size | | | | 10 | | Explain how the study size was arrived at | | | | Each partner used a dissemination strategy, depending on local possibilities (existing organisations and networks). |
| Quantitative variables | | 11 | | | See data analysis | | | |  |  |
| Statistical methods | | 12 | | | See data analysis | | | |  |  |
|  |  |  |  |  | See data analysis | | | |  |  |
|  |  |  |  |  | Missing data were less than 10% for each variable. Numbers, deviating from the total sample size are reported in the tables of the results. | | | |  |  |
|  |  |  |  |  | NA | | | |  |  |
|  |  |  |  |  | NA | | | |  |  |
| Participants | | 13* | | | Societal recognition, n=1618; professional recognition, n=2335. We reported in the discussion: Our self-selected sample with an unknown response rate might have led to a distortion of the results due to only the most motivated people participating. | | | |  |  |
|  |  |  |  |  | NA | | | |  |  |
|  |  |  |  |  | NA | | | |  |  |
| Descriptive data | | 14* | | | Results – description of research population + table 1 | | | |  |  |
|  |  |  |  |  | NA | | | |  |  |
|  |  |  |  |  |  | | | |  |  |
| Outcome data | | 15* | | |  | | | |  |  |
|  |  |  |  |  |  | | | |  |  |
|  |  |  |  |  | See results. Societal recognition (p8-11), professional recognition (p11-15) | | | |  |  |
| Main results | | 16 | | | Linear regression for the prediction of socio-economic prestige towards nurses & Logistic regression for the prediction of high professional recognition by nurses | | | |  |  |
|  |  |  |  |  | NA | | | |  |  |
|  |  |  |  |  | NA | | | |  |  |
| Other analyses | 17 | | See results section | | | | |  |  |  |
| Key results | 18 | | See discussion | | | | |  |  |  |
| Limitations | 19 | | See strengths and limitations in discussion | | | | |  |  |  |
| Interpretation | 20 | | See overall discussion | | | | |  |  |  |
| Generalisability | 21 | | See strengths and limitations in discussion | | | | |  |  |  |
| Other information | | |  |  |  |  |  |  |  |  |
| Funding | 22 | | This work was financially supported by the University Foundation from Belgium. The authors acknowledge the Foundation's generous contribution, which facilitated the publication of this research. However, the University Foundation from Belgium did not play a role in the design of the study, data collection, analyses, interpretation of the results, or the development and submission of the manuscript. | | | | |  |  |  |

*Give information separately for cases and controls in case-control studies and, if applicable, for exposed and unexposed groups in cohort and cross-sectional studies.

**Note:** An Explanation and Elaboration article discusses each checklist item and gives methodological background and published examples of transparent reporting. The STROBE checklist is best used in conjunction with this article (freely available on the Web sites of PLoS Medicine at http://www.plosmedicine.org/, Annals of Internal Medicine at http://www.annals.org/, and Epidemiology at http://www.epidem.com/). Information on the STROBE Initiative is available at www.strobe-statement.org.
